# Integrated Medicine for Chemotherapy-Induced Peripheral Neuropathy

**DOI:** 10.3390/ijms22179257

**Published:** 2021-08-26

**Authors:** Chih-Hung Tsai, Yuan-Ho Lin, Yung-Sheng Li, Trung-Loc Ho, Le Huynh Hoai Thuong, Yu-Huei Liu

**Affiliations:** 1 Graduate Institute of Integrated Medicine, China Medical University, Taichung 40402, Taiwan; seiphore@gmail.com (C.-H.T.); ed107676@edah.org.tw (Y.-H.L.); good111449@gmail.com (Y.-S.L.); 2 Department of Neurology, National Taiwan University Hospital Yunlin Branch, Yunlin 64041, Taiwan; 3 Department of Chinese Medicine of E-Da Cancer Hospital, Kaohsiung 82445, Taiwan; 4 Department of Chinese Medicine of Jiannren Hospital, Kaohsiung 811504, Taiwan; 5 International Master’s Program of Biomedical Sciences, China Medical University, Taichun 40402, Taiwan; trungloc96@gmail.com (T.-L.H.); hoaithuonglehuynh@gmail.com (L.H.H.T.); 6 Department of Medical Genetics and Medical Research, China Medical University Hospital, Taichung 40402, Taiwan; 7 Drug Development Center, China Medical University, Taichung 40402, Taiwan

**Keywords:** chemotherapy-induced peripheral neuropathy, cancer survivors, new drug development, alternative and complimentary medicines

## Abstract

Chemotherapy-induced peripheral neuropathy (CIPN) is a common side effect of typical chemotherapeutics among cancer survivors. Despite the recent progress, the effective prevention and treatment strategies for CIPN remain limited. Better understanding of the pathogenesis of CIPN may provide new niches for developing a new ideal therapeutic strategy. This review summarizes the current understanding of CIPN and current recommendations along with completed/active clinical trials and aims to foster translational research to improve the development of effective strategies for managing CIPN.

## 1. Introduction

Chemotherapy-induced peripheral neuropathy (CIPN) often occurs in cancer patients receiving neurotoxic chemotherapies. It often affects sensory neurons resulting in severe pain, which may lead to long-term morbidity in cancer survivors. Owing to the improvement in cancer survival rate, an increase in the prevalence and burden of CIPN is expected. Forty-seven percent of cancer survivors presented persistent neuropathy up to 6 years after chemotherapy completion. They exhibited altered gait patterns with slower and shorter steps, and had a 1.8-fold increase in fall risk than those without CIPN [1]. Additionally, it was reported that 12% of cancer survivors with CIPN fell within three months [2]. These observations highlight the need for an effective treatment for CIPN to improve the quality of life and safety among cancer survivors.

Currently, no treatments have been recommended to prevent CIPN. The lack of a specific target for chemotherapies is a significant challenge in CIPN management. A deeper understanding of the underlying mechanisms of how CIPN develops and progresses may help in developing novel effective strategies for prevention and treatment [3,4,5,6,7,8]. Additionally, a better way to translate the mechanistic understandings into clinical interventions, which will promote the development of new effective strategies, remains a challenge [9]. Nevertheless, an ideal study design based on known mechanisms will help in addressing the unmet medical need. This review summarizes the current understanding of CIPN and current recommendations based on completed/active clinical trials in Western medicine and alternative and complimentary medicines.

## 2. The Current Understanding of CIPN: The Pathophysiology and Molecular Mechanisms

CIPN is a common, painful, dose-limiting neurotoxic side effect of chemotherapeutics for breast, gastrointestinal, gynecologic, and hematologic cancers. Its prevalence will increase owing to the improvement in cancer survival. More than 68% of patients suffer from this condition after receiving chemotherapies [6,10,11,12,13]. Classical chemotherapeutics, including platinum analogs (cisplatin, carboplatin, and oxaliplatin), antimitotic agents (taxanes and vinca alkaloids), and proteasome inhibitors, have higher risks in the development of CIPN [14]. Typical CIPN symptoms start during the first 2 months of treatment. CIPN progresses during chemotherapy but stabilizes after the completion of treatment. However, many patients experience uninterrupted limb numbness, tightness, and pain, which influences sleep, mood, and quality of life. Although most CIPN occurs in a dose-dependent manner, other drug-specific syndromes such as paclitaxel- and oxaliplatin-induced acute neurotoxicity or cisplatin discontinuation that caused worsening neuropathy were also observed. The most common chemotherapies, their estimated cumulative dose associated with neuropathy, and the drug-specific clinical features in patients with CIPN have been summarized elsewhere [10,15,16,17,18,19,20,21,22,23,24] and in Table 1.

Because of the absence of the blood–brain barrier and excellent lymphatic drainage, the peripheral nervous system (PNS) develops CIPN much easily than the central nervous system [11,25]. Moreover, it is much easier to penetrate sensory neurons than motor neurons owing to the lesser myelination [10]. The mechanisms are complex with peripheral, spinal, and supraspinal changes, ranging from the alternation of ion channel activity to intracellular signaling systems [26,27]. Common pathological mechanisms may include mitochondrial dysfunction, imbalance in redox homeostasis, inflammation leading to apoptosis, and nerve degeneration [28]. However, drug type, cumulative dosage, clinical features, and the time course of neuropathic symptoms vary among patients. The way of administration may affect the development of CIPN. Methotrexate will be associated with neurotoxicity only with intrathecal administration [29]. Bortezomib-induced CIPN can be reduced using subcutaneous administration [22]. Genetic variations may also set a role in the gene-environment interaction, which may act as predictive CIPN biomarkers [30,31,32,33,34,35,36,37,38,39,40,41,42,43], and are one of the risk factors for developing CIPN. It is recognized that the PNS damage triggers the migration of macrophages and Schwann cells into the lesions to clean up debris, followed by the release of neurotrophic factors by Schwann cells to promote neuroregeneration. Recently, the stimulator of interferon genes-interferon type I (STING–IFN-I) signaling axis was recognized as a critical regulator of physiological nociception and a promising target for treating CIPN [44]. Galactin-3 released by Schwann cells was also reported as a critical factor to cause CIPN [45].

Specific mechanisms of neurotoxic chemotherapies vary but may highly associate with their primary roles in anticancer effects. Platinum agents, such as cisplatin and oxaliplatin, exert damage via DNA cross-linking or oxidative stress, leading to mitochondrial dysfunction and neuronal apoptosis in the dorsal root ganglia [46,47,48,49]. Moreover, oxalate metabolized from oxaliplatin prolongs the open state of the voltage-gated sodium channel, extending neuron depolarization and hyperexcitability [50]. It is noted that, unlike cancer cells, cells affected by CIPN are non-dividing. The distinct responses between the high-dividing cancer cells and non-dividing neuronal cells includes the imbalance of protepstasis, pointed a direction to simutaneously prolong neuronal cell survival via improving protein refolding by which to get chance to remove DNA adducts via DNA repair process.

Taxanes inhibit microtubule depolymerization via stabilizing GDP-bound tubulin, leading to mitotic arrest during the cell cycle G2/M phase [51]. Additionally, taxanes disrupt axonal energy supply by targeting mitochondria complexes I and II in primary afferent neurons [52,53]. Furthermore, paclitaxel induces the upregulation of toll-like receptor 4 and monocyte chemotactic protein 1 in the dorsal root ganglion, which triggers macrophage infiltration and corresponding inflammation [54]. Nevertheless, the upregulation of transient receptor potential cation channel subfamily V member 4 in the dorsal root ganglion has been linked to paclitaxel-induced neuropathic pain [55].

Unlike taxanes, vinca alkaloids prevent microtubule polymerization by binding and inhibiting tubulin-dependent GTP hydrolysis [56,57]. Vincristine-induced CIPN has been linked to the reduction of endomorphin-2 levels, thus disrupting its analgesic effect on mu-opioid receptors and subsequently leading to hypersensitivity and CIPN [58]. Additionally, chemotherapeutics-induced reactive oxygen species affect serine protease activity and afferent pain pathways [59,60]. Improved understanding of the underlying mechanisms will help in the development of new therapeutic/preventive approaches for CIPN. However, a better translation of those mechanisms into clinical benefits remains a challenge.

## 3. Current Treatment of CIPN—In the View of Western Medicine

There are no preventative treatments for CIPN [61,62]. The current primary recommended therapy for CIPN focuses on pain relief and symptom management with analgesics, antidepressants, and antiepileptics in clinical practice [63]. The first-tier choices include duloxetine, pregabalin/gabapentin, or amitriptyline [64]. Pregabalin or gabapentin structurally mimic gamma aminobutyric acid with recognized efficacy in the treatment of both epilepsy and neuropathic pain. However, unsteadiness, dizziness, edema, somnolence, and loss of concentration are the main problems [7]. Although tricyclic antidepressant amitriptyline is the gold standard for neuropathic pain, urinary retention or severe dizziness may occur in patients with benign prostate hyperplasia or elderly patients [65]. Opioids, such as tramadol or lidocaine patch, used as the second-tier choices only partially relieved neuropathic pain. The adverse effects such as nausea, dizziness, and somnolence have been observed [66,67]. Vitamin B [68,69] or vitamin E [70,71,72,73,74,75], often prescribed for neuropathic pain or diabetic polyneuropathy, showed no significant improvement in pain management. Other agents have been studied in clinical trials based on postulated effects on underlying mechanisms [7,61,66,67,76,77,78]. State-of-the-art therapies such as cryotherapy [79] or induced pluripotent stem cells or fibroblast-derived neuronal subtypes, including dorsal root ganglion neurons [80,81], remain to be evaluated. Exercises such as yoga also show benefit for alleviating CIPN [82,83,84,85]. Understanding the underlying mechanisms of dual targets of rapidly dividing cancer cells and non-dividing, post-mitotic neurons remain challenging. The current recommendations or completed/active clinical trials for CIPN are summarized in Table 2. In particular, trial specifically for plantinum—especially cisplatin alone—is rare. Lack of obvious study end point may be one reason. In addition, difficulties for specific patient enrollment may be taken into account. Breakthrough for understanding the underlying mechanisms how those plantinum drugs causes neuronal cell death, and a niche for prolonging neuron survival will help to find the critical regulatory target.

## 4. Alternative and Complementary Treatment and Prevention of CIPN

In traditional Chinese medicine (TCM), the primary pathogenesis of CIPN is related to spleen deficiency (*Pi xu* 脾虛), qi deficiency (*Qi xu* 氣虛), toxicity (*Du* 毒), stagnation (*Yu* 瘀), dampness (*Shi* 濕), and kidney deficiency (*Shen xu* 腎虛) [86]. Some herbal medicines, acupuncture, and pharmacopuncture have shown benefits in managing the disease as described below. 

### 4.1. Chinese Herbal Medicine

Goshajinkigan (GJG, Ji Sheng Shen Qi Wan (濟生腎氣丸)) has been used to treat diabetic neuropathy [87,88]. Clinical studies have indicated that GJG is effective against FOLFOX regimen- [89,90,91], oxaliplatin- [92], and paclitaxel/carboplatin-induced peripheral neuropathy [93]. However, the reproducibility of its effects is challenging [94,95,96]. Shakuyaku-Kanzo-to (SYKZT, Shao Yao Gan Cao Tang (芍藥甘草湯)) has its benefit for PNS dysfunction in paclitaxel combination therapy [97]. Ogikeishigomotsuto (AC591, Huangqi Guizhi Wuwu decoction (黃耆五物湯)) has been used to treat diabetic neuropathy [98,99]. A randomized controlled study revealed that AC591 prevents oxaliplatin-induced neuropathy without reducing its antitumor activity [100]. Ginkgo biloba (GB, Ying-Shin (銀杏)) has been used for its protective effects on nervous and circulatory systems to treat diseases, including arrhythmias, ischemic heart disease, thromboses, cancer, diabetes, and Alzheimer's disease, and cognition disorders [101]. A retrospective study revealed that GB reduces the oxaliplatin-caused intensity and duration of acute dysesthesias and yields synergistic effects for anti-tumorigenesis [102].

### 4.2. Acupuncture

Acupuncture significantly reduces CIPN, such as neuropathic symptoms (pain, tingling, and numbness), quality of life, and nerve conduction, and is considered for treatment/prevention of CIPN. However, the evidence remains to be accumulated [103,104,105]. Acupuncture might help nerve repair by increasing the limbs' blood flow [106,107]. A six-week acupuncture course improves pain, numbness, and tingling in patients with grade II CIPN [108]. A randomized controlled trial showed that acupuncture plus methylcobalamin was superior to methylcobalamin alone in providing pain relief and improving the quality of life [109].

### 4.3. Electroacupuncture

The effects of electroacupuncture on CIPN remain to be evaluated. In a four-arm randomized trial, a comparison of four different treatments, including electro-acupuncture, hydroelectric baths, Vitamin B1/B6 capsules, and placebo groups, in patients with CIPN showed no therapeutic effect of electroacupuncture [14]. Although a randomized controlled trial revealed that an eight-week course of electro-acupuncture relieves CIPN symptoms [110,111], a trial focused on preventing the symptoms of CIPN by electroacupuncture was not as good as expected [112]. Transcutaneous electrical nerve stimulation (TENS) has become an alternative for CIPN treatment; however, it requires empirical clinical evidence. Although some studies have shown efficacy in nerve regeneration and a wireless, home-based TENS may be a feasible device to relieve the symptoms of CIPN such as tingling, numbness, cramping, and pain [113], valid results were not found in a preliminary case-controlled study in clinical conditions [114]. A new approach, acupuncture-like transcutaneous nerve stimulation, applies TENS to the acupoints based on TCM theory and has shown significant improvement in neuropathic pain and numbness [115]. Scrambler therapy, another type of electrical stimulation, showed its benefit for acute or chronic CIPN [116] and quality of life [117,118]. Although a randomized phase II pilot study revealed a superior effect of TENS when compared with scrambler therapy [119], the benefit for the pain score remains to be evaluated [120]. Current completed or active acupuncture clinical trials for CIPN are summarized in Table 3.

### 4.4. Honeybee Venom Pharmacopuncture

Melittin is a 26 amino acid amphiphilic peptide that accounts for 40–50% dry weight of the venom. It is the vital pharmacological component of honeybee venom [121], with analgesic, anti-inflammatory, and anticancer effects [122]. Combined with the acupoints, pharmacopuncture is suggested to improve CIPN [123,124,125,126].

### 4.5. Challenges of TCM for CIPN

Several challenges remain for applying TCM for CIPN management. TCM syndrome plays a vital role in TCM fundamental theories. The main limit is the high difficulty of comparing alternative medicines with respect to the principles of evidence-based medicine. A precise scientific method to identify specific TCM syndrome and to consider and evaluate clinical trials will be essential. Additionally, the source, process methods, active component identification, and quality control of herbal medicine remain to be standardized. Furthermore, TCM techniques such as acupuncture, concise practitioner training, acupoint selection, deep of needle insertion, and practical protocols will be crucial. Nevertheless, TCM syndrome-specific animal models, effective chemotherapeutic agents, mode of delivery (intravenous rather than intraperitoneal injection), and adequately randomized and blinded studies are needed to represent real clinical situations [4]. 

## 5. Conclusions and Future Perspectives

CIPN is a common and persistent side effect of common chemotherapeutics. Currently, there is no intervention available for its prevention, although duloxetine has shown moderate treatment efficacy. 

Study details of biological mechanisms attributing to CIPN will be required for finding the therapeutic niches. Additionally, an ideal preclinical model will be needed to better mimic individual differences, age- and gender-dependent phenotypes of interest, and the use of standardized behavioral tests for adequately powered study designs, including appropriate controls and randomization, is needed. 

Clinical studies provide additional challenges. The intervention design, eligibility criteria selection, outcome measures and study endpoints, potential effects of an intervention on chemotherapy efficacy, and sample sizes of randomized groups based on anticipated effect size and variability are critical for research success. Systemic and multidisciplinary collaborative research ensure the development of next-generation strategies for CIPN treatment/prevention and provide benefits and better quality of life for cancer survivors suffering from CIPN.

## Figures and Tables

**Table 1:** Features of standard neurotoxic chemotherapies that cause chemotherapy-induced peripheral neuropathy (CIPN).

Type	Drug	Mechanism of CIPN	Cumulative and Dose	Incidence of CIPN	Acute Neuropathy	Chronic Neuropathy	Additional Features
Platinum-Based	Cisplatin Carboplatin Oxaliplatin	Nuclear and mitochondrial DNA damage	Cisplatin >300 mg/m^2^, Oxalipatin >800 mg/m^2^ may be needed after the first dose	Cisplatin 49–100%, Carboplatin 13–42%, Oxaliplatin 85–95%	Cold-induced dysesthesias (hand/face), Muscle cramps	Sensory neuropathy/ neuronopathy, ataxia	“Coasting”, cranial nerve involvement: hearing loss, tinnitus, ageusia, Lhermitte’s phenomenon
Taxanes	Docetaxel Paclitaxel Nab-paclitaxel Cabazitaxel Ixabepilone	Stabilization of microtubule polymers	Docetaxel ~400 mg/m^2^ Paclitaxel ~1000 mg/m^2^; doses of ≥250 mg/m^2^ may be needed after the first dose	48.2%	Taste impairment	Sensorimotor neuropathy	Occasionally cranial nerves, mononeuropathies, autonomic features, “coasting”
Vinca alkaloids	Vincristine Vinblastine Vinorelbine Vindesine	Destabilization of microtubule polymers	Vincristine >4 mg/m^2^ may be needed after the first dose	20%; Vincristine 30–40%	Taste impairment	Sensorimotor neuropathy	Occasionally cranial nerves, mononeuropathies, autonomic features, possible ‘coasting’
Brentuximab vedotin	Brentuximab vedotin	Destabilization of microtubule polymers		36–53%	Demyelinating, sensorimotor neuropathy	Autonomic myokymia	Conjugated antibody
Epothilones	Eribulin	Destabilization of microtubule polymers		25%	NS	Sensorimotor neuropathy	Conjugated antibody
Ado-trastuzumab Emtansine	Ado-trastuzumab Emtansine	Destabilization of microtubule polymers		13% after the first dose	NS	Sensorimotor neuropathy	Conjugated antibody
Proteasome inhibitor	Bortezomib Carfilzomib Ixazomib	Proteasome inhibitor			NS	Small fiber neuropathy, Severe polyradiculoneuropathy	Fewer CIPNs with subcutaneous delivery of bortezomib

Abbreviations: NS, not specified.

**Table 2:** Current clinical trials for chemotherapy-induced peripheral neuropathy (CIPN).

Study Title	Identifier	Sponsor	Phase	Chemo-Therapeutics	Cancer Type	Intervention
Drug repurposing for the prevention of chemotherapy-induced peripheral neuropathy (CIPN)	NCT04780854	Cairo University	Phase 2	Paclitaxel	NS	Metformin vs. placebo
The preliminary effects of henna on CIPN	NCT04201587	Selcuk University	NA	NS	NS	Henna application vs. control
Effect of tro19622 in the treatment of patients with chemotherapy-induced peripheral neuropathy (CIPN)	NCT00876538	Hoffmann-La Roche	Phase 2	Taxanes	NS	Olesoxime (TRO19622) vs. placebo
Niagen and persistent chemotherapy-induced peripheral neuropathy	NCT04112641	University of Iowa	Phase 2	Taxanes or Platinum	NS	Nicotinamide riboside vs. placebo capsules
Suncist: a study of calmangafodipir in healthy Japanese and Caucasian subjects	NCT03430999	Pledpharma AB	Phase 1	NA	NA	Calmangafodipir vs. placebo
Pregabalin in CIPN	NCT02394951	Washington University School of Medicine	NA	Oxaliplatin, Paclitaxel, Docetaxel, or their combinations	NS	Pregabalin vs. placebo
Preventive treatment of oxaliplatin-induced peripheral neuropathy in metastatic colorectal cancer (polar-m)	NCT03654729	Pledpharma AB	Phase 3	mFOLFOX6	Metastatic colorectal cancer	Calmangafodipir (2 dosages) vs. placebo
A study to assess the efficacy and safety of oxycodone/naloxone in Korean patients with chemotherapy-induced peripheral neuropathy (CIPN)	NCT01675531	Mundipharma Korea Ltd.	Phase 4	NS	NS	Targin (oxycodone/naloxone)
Effect of hemp-CBD on patients with CIPN	NCT04398446	Main Line Health	Phase 2	NS	Non-metastatic breast, uterine, ovarian, or colorectal cancers	Hemp-based cannabidiol vs. placebo
Preventive treatment of oxaliplatin-induced peripheral neuropathy in adjuvant colorectal cancer	NCT04034355	Pledpharma AB	Phase 3	mFOLFOX6	Colorectal cancer	Calmangafodipir vs. placebo
Ozone therapy in chemotherapy-induced peripheral neuropathy: RCT (O3NPIQ)	NCT04299893	Bernardino Clavo	Phase 2, Phase 3	NS	NS	Ozone vs. oxygen
Duloxetine and neurofeedback training for the treatment of chemotherapy induced peripheral neuropathy	NCT04560673	M.D. Anderson Cancer Center	Phase 2	NS	Hematopoietic, lymphoid cell, or solid malignant neoplasms	Duloxetine vs. neurofeedback training vs. their combination
Study of nicotine for pain associated with chemotherapy-induced peripheral neuropathy	NCT04468230	Virginia Commonwealth University	Phase 2	NS	NS	Nicotine transdermal patch
Menthol in neuropathy trial (MINT)	NCT04276727	University of Edinburgh	Phase 2	NS	NS	Menthol vs. placebo
Minocycline hydrochloride in reducing chemotherapy-induced peripheral neuropathy and acute pain in patients with breast cancer undergoing treatment with paclitaxel	NCT02297412	Academic and Community Cancer Research United	Phase 2	Paclitaxel	Breast cancer	Minocycline hydrochloride vs. placebo
High dose inorganic selenium for preventing chemotherapy-induced peripheral neuropathy	NCT04201561	Seoul National University Hospital	Phase 3	Paclitaxel	Response evaluation criteria in solid tumors (RECIST), or gynecologic, epithelial ovarian, fallopian, or primary peritoneal cancers	Sodium selenite pentahydrate vs. vehicle vs. standard care
Chemotherapy-induced peripheral neuropathy-essential oil intervention	NCT03449303	Augusta University	NA	NS	Breast cancer	Eoi (10% dilution of *Curcuma longa*, *Piper nigrum*, *Pelargonium asperum*, *Zingiber officinale*, *Mentha* X *piperita*, and *Rosmarinus officinalis* Ct. *Cineole* in (*Simmondsia chinensis*) vs. placebo (*Simmondsia chinensis*)
The role of transient receptor potential channels in chemotherapy-induced peripheral neuropathic pain	NCT04415892	Universitaire Ziekenhuizen Leuven	NA	Paclitaxel or Oxaliplatin	NS	Cinnamaldehyde and capsaicin
Cannabinoids for taxane-induced peripheral neuropathy	NCT03782402	New York State Psychiatric Institute	Phase 2	Paclitaxel or Docetaxel	Breast cancer	Cannabinoids of various strengths
N-acetyl cysteine effect in peripheral neuropathy in cancer patients	NCT03492047	Ain Shams University	Phase 1, Phase 2	Paclitaxel	Breast cancer	N-acetylcysteine (low vs. high dose) vs. standard care
Lidocaine versus duloxetine for the prevention of taxane-induced peripheral neuropathy in breast cancer patients	NCT04732455	Gamal Mohamed Taha Abouelmagd	NA	Taxanes	Breast cancer	Lidocaine vs. vehicle vs. duloxetine
The potential protective role of venlafaxine versus memantine in paclitaxel-induced peripheral neuropathy	NCT04737967	Mendel AI	Phase 2, Phase 3	Paclitaxel	NS	Venlafaxine vs. memantine
NR in chemo-induced peripheral neuropathy	NCT03642990	Donna Hammond	Phase 2	Paclitaxel	Metastatic breast cancer	Nicotinamide riboside
NR in chemo-induced peripheral neuropathy	NCT03642990	Donna Hammond	Phase 2	Platinum	Platinum-resistant recurrent ovarian, peritoneal, endometrial, fallopian tube, or head and neck cancers	Nicotinamide riboside
Duloxetine in treating peripheral neuropathy caused by chemotherapy in patients with cancer	NCT00489411	Alliance for Clinical Trials in Oncology	Phase 3	Taxanes or Platinum	NS	Duloxetine hydrochloride vs. placebo
Vitamin e in preventing peripheral neuropathy caused by chemotherapy in patients receiving chemotherapy for cancer	NCT00363129	Alliance for Clinical Trials in Oncology	Phase 3	Taxanes or Platinum	NS	Vitamin E vs. placebo
Lamotrigine in treating peripheral neuropathy caused by chemotherapy in patients with cancer	NCT00068445	Alliance for Clinical Trials in Oncology	Phase 3	Taxanes, Platinum, Vinca Alkaloids	NS	Lamotrigine vs. placebo
Clinical study on acetyl-l-carnitine	NCT01526564	Lee's Pharmaceutical Limited	Phase 3	Taxoids, Satraplatin and Vincristine	NS	Acetylcarnitine vs. placebo
Gabapentin in treating peripheral neuropathy in cancer patients undergoing chemotherapy	NCT00027963	Alliance for Clinical Trials in Oncology	Phase 3	Taxanes, Platinum, or Vinca alkaloids	NS	Gabapentin vs. placebo
Baclofen-amitriptyline hydrochloride-ketamine gel in treating peripheral neuropathy caused by chemotherapy in patients with cancer	NCT00516503	Alliance for Clinical Trials in Oncology	Phase 3	NS	Chronic myeloproliferative disorders, leukemia lymphoma, lymphoproliferative disorder, multiple myeloma and plasma cell neoplasm, myelodysplastic syndromes, myelodysplastic/myeloproliferative neoplasms	Baclofen/amitriptyline/ketamine gel vs. placebo
Fingolimod in treating patients with chemotherapy-induced neuropathy	NCT03943498	Mayo Clinic	Early Phase 1	NS	NS	Fingolimod vs. Fingolimod hydrochloride

Abbreviations: NA, not applicable; NS, not specified.

**Table 3:** Current complete or active acupuncture clinical trials for chemotherapy-induced peripheral neuropathy (CIPN).

Study Title	Identifier	Sponsor	Phase	Chemotherapeutics	Cancer Type	Intervention
Oral Cryotherapy Plus Acupressure and Acupuncture Versus Oral Cryotherapy for Decreasing Chemotherapy-Induced Peripheral Neuropathy From Oxaliplatin-Based Chemotherapy in Patients With Gastrointestinal Cancer	NCT04505553	University of Washington	Phase 2 Pilot Study	Oxaliplatin-Based Chemotherapy	Gastrointestinal Cancer	Oral cryotherapy vs. oral cryotherapy plus acupuncture/acupressure
Acupuncture in Reducing Chemotherapy-Induced Peripheral Neuropathy in Participants With Stage I-III Breast Cancer	NCT03505671	Wake Forest University Health Sciences	NA	NS	Breast Cancer	Acupuncture vs. standard care
Acupuncture for Peripheral Neuropathy Induced by Paclitaxel in Early Stage Breast Cancer	NCT04461977	Instituto Brasileiro de Controle do Cancer	NA	NS	Breast cancer (stages I, II, III)	Acupuncture vs. sham acupuncture
Acupuncture for CIPN in Breast Cancer Patients	NCT02615678	Southern California University of Health Sciences	NA	NS	Breast Cancer	Before and after acupuncture
Integrative Medicine for Chemotherapy-Induced Peripheral Neuropathy	NCT03290976	The Chaim Sheba Medical Center	NA	Taxanes	1. Female patients with breast or gynecological cancers	Single vs. multi-modality acupuncture vs. standard care
Integrative Medicine for Chemotherapy-Induced Peripheral Neuropathy	NCT03290976	The Chaim Sheba Medical Center	NA	NS	2. Patients of either gender with hematological malignancies	Single vs. multi-modality acupuncture vs. standard care
Standard Care Alone or With Acupuncture for CIPN in Breast Cancer and Multiple Myeloma (ACUFOCIN)	NCT02275403	The Christie NHS Foundation Trust	Phase 2	NS	Breast cancer, multiple myeloma, gastrointestinal cancer, or gynecological cancer	Acupuncture vs. standard care
The Use of Acupuncture for Treatment of Chemotherapy-induced Peripheral Neuropathy (CIPN)	NCT02309164	University of Sao Paulo	NA	NS	NS	Acupuncture vs. standard care
Evaluation of the Efficacy of Acupuncture in Chemotherapy-Induced Peripheral Neuropathy	NCT03626220	China Medical University Hospital	NA	NS	Breast cancer	Acupuncture vs. sham acupuncture
The Effectiveness and Cost-Effectiveness of Acupuncture in Managing Chemotherapy-induced Peripheral Neuropathy	NCT02553863	The Hong Kong Polytechnic University	NA	NS	Lung, breast, gynecological, or head & neck cancers, or colorectal cancer (stage I, II, III).	Acupuncture vs. standard care
Efficacy of Acupuncture on Chemotherapy-Induced Peripheral Neuropathy	NCT04739631	Taipei Veterans General Hospital, Taiwan	NA	Taxanes (paclitaxel or docetaxel), platinum (cisplatin, oxaliplatin, carboplatin)	NS	Acupuncture vs. sham acupuncture
Testing the Effects of Transcutaneous Electrical Nerve Stimulation (TENS) on Chemotherapy-Induced Peripheral Neuropathy (CIPN)	NCT04367480	University of Rochester NCORP Research Base	NA	NS	NS	TENS
Feasibility Study for Electroacupuncture for Chemotherapy- Induced Peripheral Neuropathy (CIPN)	NCT04092764	H. Lee Moffitt Cancer Center and Research Institute	NA	Taxanes or Platinum-Based	NS	Electroacupuncture vs. NeuroMetrix vs. Rydel-Seiffer tuning fork
Acupuncture for Chemotherapy-induced Peripheral Neuropathy	NCT03582423	Hong Kong Baptist University	NA	Eight cycles of adjuvant oxaliplatin-based chemotherapy	Stage II–III colorectal cancer	Electroacupuncture vs. sham acupuncture
Scrambler Therapy in the Treatment of Chronic Chemotherapy-Induced Peripheral Neuropathy	NCT02111174	Sidney Kimmel Comprehensive Cancer Center at Johns Hopkins	NA	NS	NS	Scrambler therapy vs. sham therapy

Abbreviations: NA, not applicable; NS, not specified; TENS, Transcutaneous Electrical Nerve Stimulation.
